# Dietary and Oral Hygiene Behaviors Associated with Prevalent Caries Status in School-Aged Children of Northern Italy

**DOI:** 10.3390/nu18091416

**Published:** 2026-04-29

**Authors:** Virginia Troiani, Edoardo Ratti, Daniel Gonnella, Maria Cristina Panzeri, Paola Palestini, Emanuela Cazzaniga

**Affiliations:** 1 School of Medicine and Surgery, University of Milano-Bicocca, Via Cadore 48, 20900 Monza, Italy; virgi.troiani@gmail.com (V.T.); e.ratti9@campus.unimib.it (E.R.); danielgonnella.11@gmail.com (D.G.); mariacristina.panzeri@unimib.it (M.C.P.); paola.palestini@unimib.it (P.P.); 2 Master’s Programme in Applied Food and Nutritional Sciences, School of Medicine and Surgery, University of Milano-Bicocca, 20900 Monza, Italy

**Keywords:** children, oral health, dental caries, educational program, nutrition, prevention

## Abstract

**Background/Objectives:** Unhealthy dietary behaviors and suboptimal oral hygiene practices remain common among Italian children, potentially affecting both nutritional and oral health. Dental caries, a preventable yet highly prevalent condition in pediatric populations, has a multifactorial etiology in which lifestyle factors play a key role. This study aimed to assess the prevalence of dental caries, dietary habits, and oral hygiene behaviors in school-aged children in Lombardy, and to identify factors associated with prevalent caries status. **Methods:** A cross-sectional study was conducted on 307 schoolchildren aged 9–10 years from ten schools in Northern Italy. Oral health status was evaluated through the plaque index and the DMFT/dmft index during school-based dental examinations. Dietary habits, lifestyle, and oral hygiene practices were collected through structured questionnaires. A mixed-effects logistic regression model was developed to explore potential associations between variables and prevalent caries status. **Results:** The dietary patterns, weight status, oral hygiene behaviors, and oral health conditions were generally consistent with the national data. Higher plaque index, skipping breakfast, consuming mid-morning snacks, and parental reports of previous caries experiences were retained in the final model. Internal validation suggested reasonable discriminatory ability overall, whereas calibration shows heterogeneity across schools. **Conclusions:** The findings highlight suboptimal dietary and oral hygiene behaviors among Lombardy schoolchildren and confirm their association with dental caries. Lifestyle-related factors, particularly oral hygiene practices and eating patterns, showed a relevant association with prevalent caries status in the analyzed sample. These results underscore the need for targeted preventive strategies integrating nutritional education and oral health promotion in pediatric populations.

## 1. Introduction

Dental caries is the localized deterioration of dental hard tissues by acidic products from bacterial fermentation of dietary carbohydrates [1]. According to the National Epidemiological Survey on the Dentoperiodontal Conditions of 4- and 12-Year-Old Children, conducted in 2005 among the Italian population, 20.6% of 4-year-old children and 42.3% of 12-year-old children presented with dental caries [2]. Although various epidemiological studies have shown a significant reduction in the prevalence of dental caries among children and adolescents over the past two decades in industrialized countries, this remains one of the most common noncommunicable childhood diseases [1,3,4]. Dental caries is a multifactorial disease; its development is related to lifestyle and to several behavioral factors like poor oral hygiene and poor dietary habits [1,5]. Notably, the consumption of sugars seems to be an important cause in the etiology of caries [3,4,6,7,8]: no level of sugar consumption can be regarded as risk-free for the development of dental caries [7], and both the amount and frequency of sugar consumption are risk factors [4,6,7]. Dental caries is a widespread disease despite being both preventable and treatable. Managing this condition is important not only because it causes significant physical and psychological discomfort in children but also due to its recurrent nature in adulthood [1,3]. Moreover, it represents a considerable burden on the healthcare system in industrialized countries [4].

Another manageable condition causing significant global concern is the excessive accumulation of adipose tissue, often resulting in overweight or obesity; this is a multifactorial condition resulting from the interplay of individual and environmental factors [9,10]. Nowadays, nutritional imbalances associated with a diet that exceeds physiological needs are becoming more common. In the last decade a rise has been observed in the consumption of snacks and, more notably, sugar-sweetened beverages, including soft drinks and fruit juices. Insufficient intake of fruits and vegetables goes hand in hand with the increasing consumption of processed foods that are high in salt, lipids and simple sugars, especially among children [11,12,13]. In Italy, unhealthy dietary habits and lifestyles are still highly prevalent and have a negative impact on children’s health [11].

Obesity and dental caries share several common features. Both are chronic noncommunicable diseases with a multifactorial etiology that is influenced by individual and environmental risk factors, and both are largely preventable and manageable conditions.

These similarities suggest that the two conditions may share common environmental determinants, particularly dietary habits and lifestyle behaviors. While the genetic and biological susceptibilities differ between the two conditions, numerous environmental determinants show significant overlap, particularly in relation to the risk factors and protective behaviors implicated in their pathogenesis. Among the most relevant environmental determinants are dietary patterns, eating behaviors, and lifestyle factors, including adherence to the Mediterranean diet, breakfast skipping and frequent consumption of unhealthy snacks [14,15,16,17]. Good adherence to the Mediterranean diet means not only the consumption of nutrient-rich healthy foods but also the avoidance of behaviors that are considered to be detrimental to health, like low intake of milk, dairy products, fruits and vegetables, skipping breakfast and the frequent consumption of ultra-processed snacks and sugar-sweetened beverages (SSBs), which are associated with high intake of saturated fats and free sugars. Several foods and beverages, including dairy products, fruits, vegetables, whole grains, nuts, and certain polyphenol-rich beverages such as coffee, tea, and cocoa, have been suggested to exert protective effects against both dental caries and excess body weight [18,19,20,21,22]. In contrast, foods that are rich in simple sugars—such as sugary snacks, ultra-processed foods, candies, SSBs, energy drinks, and fruit juices with added sugars—are considered to be cariogenic and represent recognized dietary risk factors for the development of overweight and obesity [12,14,18,23,24].

Therefore, the aim of this study was to investigate the prevalence of dietary and oral habits in 9/10-year-old children in Lombardy and to explore, through multivariable modeling, which variables were associated with prevalent caries status in the analyzed sample.

## 2. Materials and Methods

This cross-sectional study is part of the “Denti sani e ben nutriti” project, conducted by the University of Milano-Bicocca since 2017 [25]. The present analysis focused on fourth-grade children attending ten primary schools in Northern Italy: Monza Buonarroti (Monza), Monza Manzoni (Monza), Monza Citterio (Monza), Desio Collegio Di Rosa (Monza), Madone (Bergamo), Merate Sartirana (Lecco), Villa D’Adda (Bergamo), Mapello (Bergamo) Bonate Sotto (Bergamo), and Brembate Sopra (Bergamo). The schools participating in the 2024 educational program were located in Lombardy and were selected on a convenience basis. Originally, eleven schools were included in the 2024 educational program; however, one school (Merate Montello) contributed no analyzable observations after the exclusion procedure and was therefore not included in the final analyzed sample (see Section 3.1). Educational sessions on oral hygiene and nutrition were carried out between January and June 2024. During these sessions, the children underwent oral examinations to assess the DMFT/dmft index and the plaque index, following the O’Leary method [26]. Data regarding dietary and oral hygiene habits were collected through specifically designed questionnaires and the validated KIDMED questionnaire [27], administered to both parents and children. The “oral hygiene habits” questionnaire—administered to the parents only—consisted of sixteen questions about the children’s oral habits and oral health (IG11–IG16). The KIDMED (K1–K14) questionnaire was administered to both parents (at home) and children (at school). The “dietary habits” questionnaire, administered to the children at school, consisted of seventeen questions about diet and oral hygiene (SNACK1–SNACK17).

### Statistical Analysis

Continuous variables were summarized using means and standard deviations, and categorical variables using absolute and relative frequencies. The primary outcome for the multivariable analysis was prevalent caries status at the time of examination, defined by dichotomizing DMFT/dmft as 0 versus ≥ 1. This binary endpoint was chosen to capture the presence of any prevalent caries experience because, from a clinical and preventive perspective, even a single carious lesion in a 9–10-year-old child was considered sufficient to warrant attention. Accordingly, modeling DMFT/dmft as 0 versus ≥ 1 also provided a more stable basis for model development given the modest sample size and the highly skewed distribution of DMFT/dmft. To account for clustering of children within schools, a mixed-effects logistic regression model with random intercept for school was fitted. Candidate variables (Box 1) were identified a priori on substantive grounds from the available dietary, oral hygiene, and demographic information and were then reduced through a pre-specified screening strategy aimed at limiting instability and redundancy in a modest-sized dataset. Continuous variables were standardized before modeling, and the linearity with the transformed outcome was graphically assessed. Variables were excluded before multivariable modeling if they showed negligible information for the outcome (Information Value, IV < 0.02) or problematic collinearity (variance inflation factor >= 5 or tolerance ≤ 0.2) [28]. Among the remaining variables, variable selection was performed by backward elimination based on the Akaike information criterion (AIC) [29]. Only main effects were considered (no interaction terms). The final set of selected variables is reported in Box 1.

Given the limited sample size, the full analyzed dataset was used for model development. To quantify internal validity, evaluate the stability of the selected variables, and estimate optimism-corrected performance, 500 bootstrap resamples were generated. Given the limited number of clusters, an individual-level bootstrap was used, resampling children within schools in each bootstrap sample. Variable stability was summarized by the frequency with which each variable and the final overall model specification was retained across bootstrap replicates. Model performance was assessed in terms of discrimination and calibration (Box A1). Discrimination and calibration were assessed separately within each school using predictions incorporating the school-specific random intercept and then pooled across schools using a random-effects meta-analysis [30]. All performance measures were optimism-corrected using the 0.632 method [31]. Pooled optimism-corrected estimates were obtained by conducting the random-effects meta-analysis on the school-specific optimism-corrected performance measures. Ninety-five percent confidence intervals (95% CIs) were computed using the location-shifted approach [32]. Due to the paucity of missing data on the final sample, complete case analysis was considered.

Because variable selection was partly data-driven, only standard errors for model coefficients are reported as *p* values are not valid in this setting. All analyses were performed using SAS 9.4M9.

Box 1 **Variables.**

Candidate Variables

Sex, parents’ nationality, parents’ education, SNACK_3, SNACK_4, SNACK_5, SNACK_6, SNACK_7, SNACK_12,

SNACK_14, K1, K3, K6, K12, K13, K14, K16, IG7, IG12, *has the child ever been to the dentist?* BMI, Cole class, plaque index

Selected Effects

SNACK_3, plaque index, K1, K12, IG12

## 3. Results

### 3.1. Study Population and Sample Characteristics

A total of 497 participants from eleven schools were initially enrolled in the study, with the student distribution across schools ranging from 6.0% to 14.1% of the total sample (Table 1).

Eighty-two questionnaires were excluded from the final analysis (Table 2), including 32 (39.0%) due to a lack of parental consent and 50 (61.0%) judged to be falsified. All the falsified questionnaires originated from a single school, Merate Montello, whereas non-consent exclusions were more evenly distributed across schools (Table 2).

An additional 107 students were excluded due to absence on the day of data collection or lost questionnaires (Table 3). Specifically, 11 students (10.3%) were absent, and 96 (89.7%) questionnaires were lost (Table 3).

After the exclusion procedure, only one student remained from Merate Montello; therefore, this school was not included in the final analyzed sample.

**Figure 1:**
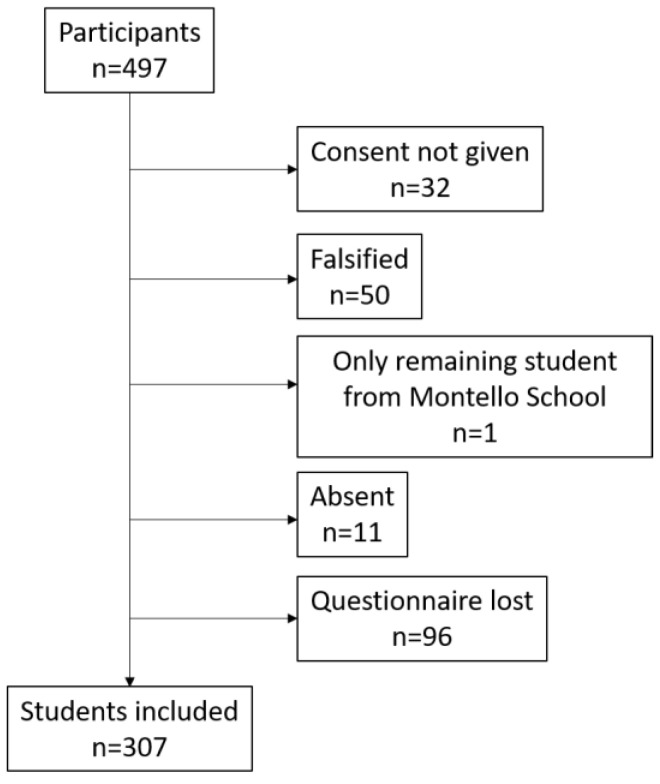
Flow chart of participant selection showing the number of subjects included after the exclusion procedure.

The final analytical sample comprised 307 children from ten schools (mean age 9.2 years, SD 0.42; median 9.0; range 9.0–12.0), with balanced gender distribution (50.5% males) (Table 4, Figure 1).

Most children were Italian (96.0%), with 4.0% of foreign nationality (seven missing). Regarding parental nationality, 81.2% had both Italian parents, 12.2% had both foreign parents, and 6.6% had only one Italian parent (four missing). Parental education was primarily high school level (49.3%), followed by university degree (31.6%) and middle school or lower (19.1%) (three missing) (Table 5).

According to the Cole classification [33,34,35], most of the children are normal weight (72.6%) and almost two in ten children are overweight or obese (21.2% and 3.3%), with a marked difference among schools, ranging from 4.8% overweight/obese children in Merate Sartirana to 40.8% overweight or obese children in Monza Citterio. The Mediterranean diet adherence index [27] averaged 5.0 (SD 2.47; median 5.0; range −1.0 to 11.0) in parents (n = 306) and 5.6 (SD 2.59; median 6.0; range −1.0 to 11.0) in children (n = 307). Categorically, 23.1% of the children showed low adherence, 53.1% moderate adherence, and 23.8% high adherence (Table 5).

For completeness regarding the representativeness of the analyzed sample, we report in Appendix A (Table A1) the demographic characteristics of the children of Merate Montello school with falsified questionnaires as these were the only excluded participants for whom questionnaires were available. Age was consistent with the included sample (mean 9.3 years, SD 0.47; median 9.0; range 9.0–10.0), and sex was balanced (46.9% male, 53.1% female). However, the proportion of missing data was very high for the remaining variables, including child nationality, parental nationality, parental education, and weight status.

### 3.2. Nutritional Habits

Most of the children eat breakfast before school (91.5%) and have a mid-morning snack at school (86.6%), whereas, on the weekend, most of the children eat breakfast (94.8%) but do not have a mid-morning snack (74.2%). Almost all the children have an afternoon snack after school (92.2%). Most of the children in the sample eat both sweet and salty food as snacks (42.1%) and drink other beverages (52.5%) instead of juice (39.9%), milk (5.0%) and sugar-sweetened beverages (2.6%). Notably, 62.4% of the children eat fruit as a snack and 41.0% eat yogurt or drink milk. Nearly 50% of the children drink a bottle of water during school time (44.7%), while almost one in three children drink less than one bottle (36.2%). Further, 87.6% bring snacks from home, and more than 50% choose them by themselves (54.7%).

Many of the children had nutritional education classes in current or previous years (71.7%) (Table 6).

Most of the children eat one fruit daily (72.6%), while less than half of the sample eat fruit twice in a day (42.3%). Similarly, 69.1% of the children eat vegetables daily, but only 35.8% of them eat vegetables twice daily.

More than half of the sample reported consuming pasta or rice at least five times per week (76.9%), eating legumes more than once weekly (58.6%), and using olive oil as the primary seasoning at home (84.7%). In contrast, fewer than half of the children consumed fish or dried fruit at least 2–3 times per week (39.7% and 43.3%, respectively) or reported eating yogurt or cheese twice daily (43.6%). Additionally, 13.4% of the children visited fast-food restaurants more than once per week, while nearly three in ten reported consuming sweets or candy several times per day (29.3%).

As reported in Table 6, the majority of the children reported regularly consuming breakfast (90.9%). The most commonly consumed breakfast items were snacks or cookies (70.4%), followed by milk or other dairy products (61.2%), and cereals, bread, or rusks (50.6%) (Table 7).

### 3.3. Oral Hygiene Habits and Dental History

Based on the self-reported questionnaire completed by the children in the classroom (Table 6), 63.2% of the sample reported brushing their teeth twice a day, 20.8% more than twice a day, 15.6% once a day, and only 0.3% reported never brushing their teeth. Most children reported always brushing their teeth after breakfast before going to school (74.6%), although this percentage decreased on weekends (57.2%). In addition, 69.4% of the children reported brushing their teeth before going to bed, 17.9% reported doing so frequently, 6.8% only “a little”, and 5.9% reported never brushing their teeth before bedtime.

According to the parent-completed questionnaire on children’s oral hygiene habits (Table 8), the mean age at the first dental visit was 5.8 years (SD = 1.62), with slight differences observed among the schools.

Overall, 95.7% of the parents reported that their children brush their teeth daily, and 87.5% indicated that their children are generally autonomous in performing oral hygiene practices. Approximately 80% of the children brush their teeth both in the morning and in the evening; however, this proportion was markedly lower in two schools, Monza Citterio (54.5%) and Madone (54.2%).

Regarding home oral hygiene tools, 87.2% of the parents reported that their children use an age-appropriate toothbrush. Nevertheless, 10.7% declared the use of fluoride-free toothpaste, and 16.6% reported the use of toothpaste not specifically formulated for children of this age.

Regular dental attendance was common: 46.1% of the children visited the dentist more than twice per year. This result is consistent with the responses reported by the children in the questionnaire completed in class (Table 9).

The primary reason for the first dental visit was routine check-up (68.5%), although visits due to pain (14.3%) and trauma (5.0%) were also reported.

With respect to dental caries, 47.9% of the children had experienced at least one carious lesion. Tooth loss due to caries was reported in 10.2% of cases. Additionally, 9.9% of the children had lost deciduous teeth prematurely, and 1.0% had lost permanent teeth, with considerable variability observed across the schools (Table 8).

Oral health assessments conducted during school-based dental examinations showed a mean DMFT/dmft of 0.7 (SD = 1.26), with marked variability across the schools. The mean DMFT/dmft ranged from 0.2 (SD = 0.84) in Desio Collegio de Rosa to 1.2 (SD = 1.90) in Madone. Overall, 30% of the children presented with at least one decayed tooth. The plaque index was 80%, with no significant differences observed between the schools.

### 3.4. Multivariable Regression Analysis

In the mixed-effects logistic regression model with a random intercept for school, the outcome was the probability of prevalent caries status, defined as DMFT/dmft >= 1 at the time of examination. Among the a priori candidate variables, the following were excluded based on the pre-specified screening criterion (IV ≤ 0.02): sex, parents’ nationality, SNACK_4, SNACK_5, SNACK_12, K3, K6, K13, K14, and K16. Regression coefficients for the variables retained after backward elimination are reported on the logit scale. Positive values indicate a higher predicted probability of DMFT ≥ 1, and negative values indicate a lower predicted probability. The reference (baseline) categories for the categorical variables were: having a mid-morning snack at school = yes (SNACK_3), consuming fruit every day = yes (K1), usually having breakfast = yes (K12), and parental report that the child has ever had cavities = yes (IG12). Therefore, each reported coefficient represents the contrast with these baseline categories. Because variable selection was partly data-driven, *p*-values are not valid, and the results are presented descriptively in terms of estimated coefficients and their standard errors (Table 10).

Higher plaque index values were associated with a markedly higher probability of prevalent caries status, suggesting that the plaque index may act as a proximal marker of prevalent caries status in this sample. In addition, not usually having breakfast (K12 = no) was associated with a higher probability of prevalent caries status compared with children who usually eat breakfast.

Conversely, not having a mid-morning snack at school (SNACK_3 = no) was associated with a lower probability of prevalent caries status compared with children who do have a snack at school. Finally, when parents reported that a child had never had cavities (IG12 = no), the probability of DMFT ≥ 1 was substantially lower than when parents reported a previous history of cavities, indicating that past caries experience is strongly associated with prevalent caries status of the sample. The coefficient for not consuming fruit every day (K1 = no) was negative in the selected model, suggesting a lower probability of prevalent caries status relative to the baseline (K1 = yes). This finding should be interpreted descriptively within the exploratory nature of the multivariable modeling process rather than as a direct etiologic effect. Importantly, this estimate appears imprecise (large variability/standard error), which further supports a cautious interpretation.

**Figure 2:**
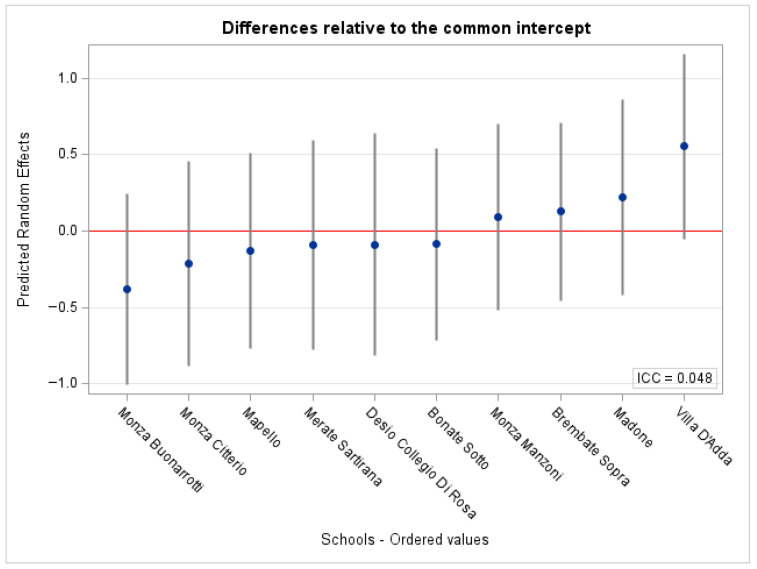
School-specific random intercepts from the mixed-effects logistic regression model for prevalent caries status (DMFT ≥ 1). Points represent estimated deviations from the common intercept (red line at 0), and vertical bars indicate uncertainty around each estimate. Schools are ordered by the estimated random intercept. ICC = Interclass Correlation Coefficient.

**Figure A1:**
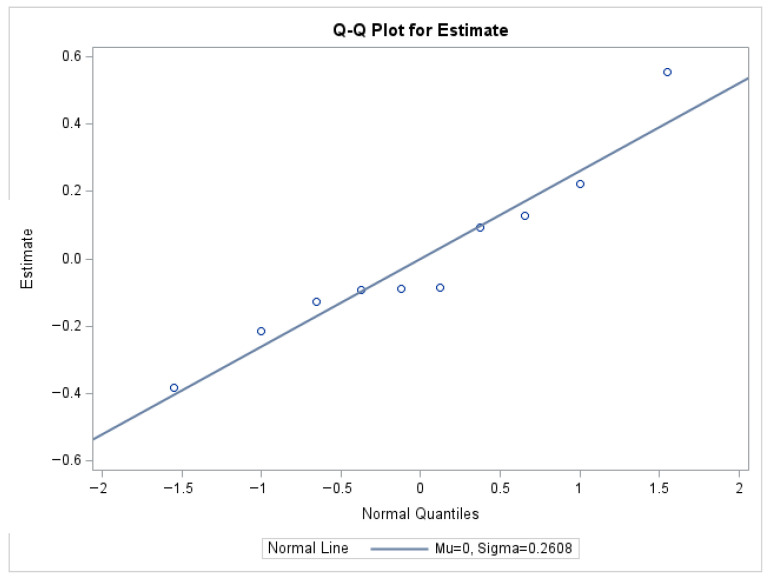
Diagnostic plot to check normality of random effects.

Figure 2 shows the school-specific random intercepts, i.e., how each school’s baseline probability of prevalent caries status differs from the overall average after accounting for the child-level variables. The schools below the red line have a lower predicted baseline probability of prevalent caries status than average, whereas those above have a higher predicted baseline probability. Overall, the differences between the schools are low after accounting for relevant covariates (ICC = 0.048). A graphical check provides a modest departure from the normality assumption of the predicted random effects (Figure A1).

**Figure 3:**
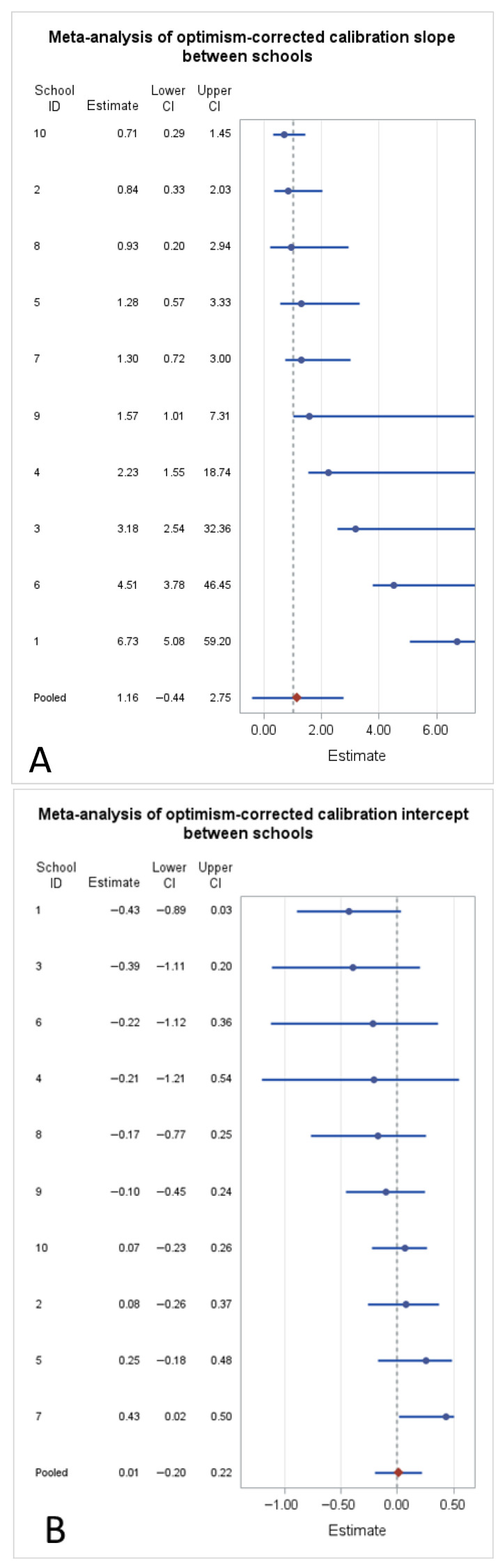

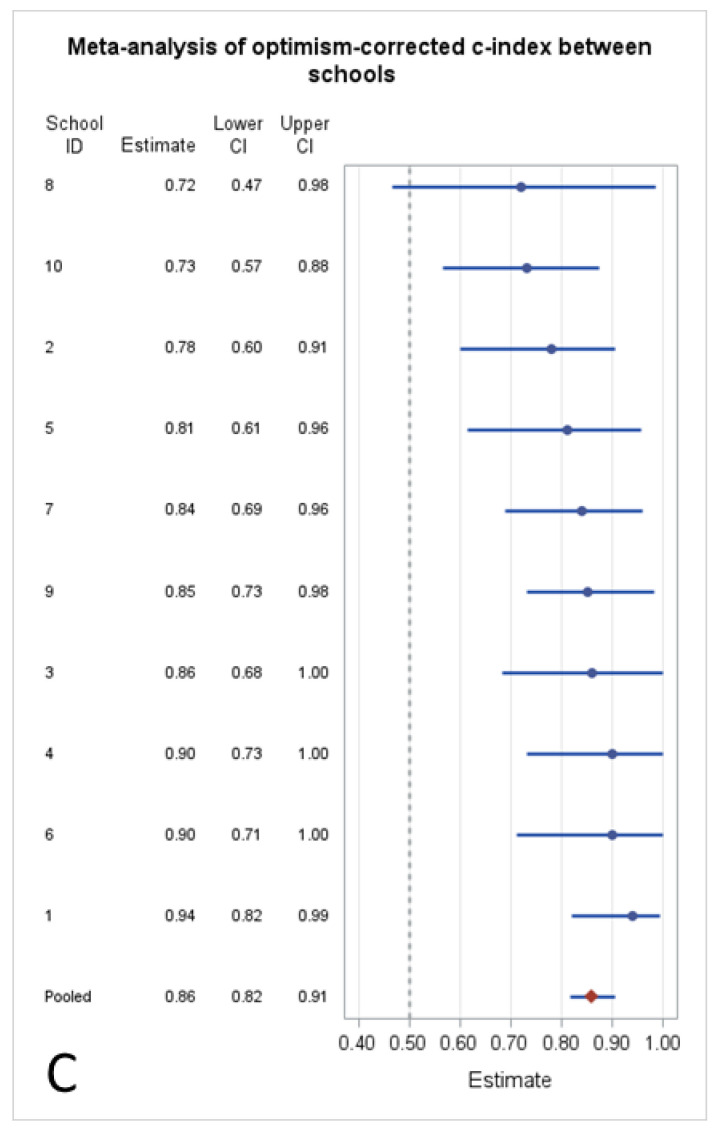
Forest plots of the optimism-corrected performance of the mixed-effects logistic model (random intercept for schools) across schools. Panels report the calibration slope (**A**), calibration intercept (**B**), and the c-index (AUC) (**C**), with point estimates and corresponding confidence intervals for each school (School ID) and the pooled estimate (“pooled”). The dashed vertical line indicates the reference value: 1 for the calibration slope (ideal calibration), 0 for the calibration intercept (no systematic over-/under-prediction), and 0.5 for the c-index (chance-level discrimination). School IDs correspond to: 1 = Monza Buonarroti, 2 = Monza Manzoni, 3 = Monza Citterio, 4 = Desio Collegio Di Rosa, 5 = Madone, 6 = Merate Sartirana, 7 = Villa D’Adda, 8 = Mapello, 9 = Bonate Sotto, and 10 = Brembate Sopra.

An internal validation suggested that the selected model had a reasonable ability to distinguish children with and without prevalent caries status (pooled c-index = 0.86). In contrast, the calibration results were more heterogeneous. The pooled optimism-corrected calibration intercept is close to 0 (no systematic over- or under-prediction on average), while the calibration slope is close to 1 in the pooled analysis, suggesting that the individual predicted probability of prevalent caries status is not markedly over-confident or overly shrunk overall. Nevertheless, the forest plots show marked within-school heterogeneity of the performance measures. In particular, the calibration intercept (Figure 3B) appears relatively consistent across the schools and close to the ideal value, suggesting limited systematic over- or under-prediction on average. In contrast, the calibration slope (Figure 3A) shows substantial heterogeneity and, for some schools, point estimates well above 1 with very wide confidence intervals. This pattern indicates that the selected multivariable model has less stable calibration at the school level and underestimated prevalent caries status in some schools.

With respect to model stability, the variables included in the final specification (SNACK_3, IP_new, K1, K12, and IG12) were among the most frequently retained across the 500 bootstrap replicates (Table A1). However, the exact model specification was less stable, ranking fifth among the most frequently selected combinations of variables across the bootstrap samples (Table A2).

## 4. Discussion

Although dental caries and obesity are largely preventable and treatable diseases—being associated with modifiable hygienic and dietary behaviors—both conditions continue to represent major global public health concerns, particularly within the pediatric population [11,14,36,37,38]. Despite the growing body of evidence supporting preventive strategies, the interventions implemented to date have shown limited effectiveness in reducing their prevalence. Notably, before 2022, no country had yet succeeded in reaching the target established by the World Health Assembly of halting the rise in adult obesity prevalence by 2025, and the recent trends suggest increasingly early onset of excess body weight among children [11,38].

Evidence from the “National Epidemiological Survey on dental and periodontal conditions among children aged 4 and 12 years” highlighted a persistent burden of oral diseases. The mean value of the DMFT/dmft index among 12-year-olds was 1.09, exceeding both the preventive target set by the World Health Organization for 2010 (DMFT < 1) and the more ambitious goal established for 2020 (DMFT < 0.5). Moreover, the proportion of caries-free 12-year-old children was 57.71%, considerably lower than the WHO target of 80% for 2020, further emphasizing the need for strengthened preventive efforts [2].

In this context, school-based educational initiatives may represent a valuable strategy for promoting early adoption of healthy behaviors. The project “Healthy and well-nourished teeth (Denti sani e ben nutriti)”, conducted by the University of Milano-Bicocca since 2017 [25], was designed to address this need by providing educational sessions for children attending preschool and primary schools. Through these activities, children were introduced to the basic principles of healthy nutrition and appropriate oral hygiene practices to be implemented at home, with a particular focus on the role of prevention in reducing the risk of both conditions.

The analysis of the sample of 307 children showed that approximately 70% of the children were classified as normal weight, whereas slightly more than 20% were classified as overweight or obese overall, with marked differences between schools. These findings slightly differ from those reported in the most recent survey of the OKkio alla SALUTE surveillance system, according to which, at the national level, 28.8% of school-aged children have body weight excess. The lower prevalence observed in the present sample may be explained by the fact that the survey was conducted in schools located in Lombardy and therefore does not fully reflect the well-known north–south gradient in childhood overweight and obesity observed in Italy. Indeed, the regional data differ only slightly from those observed in the present sample: in Lombardy, 22.8% of children are affected by excess body weight. Another possible explanation for this difference lies in the characteristics of the study population. The OKkio alla SALUTE surveillance system collects data from children attending the third year of primary school, whereas the present investigation involved students in the fourth year [11].

With regard to dietary patterns, more than 20% exhibited poor adherence to the Mediterranean diet and only approximately one fifth of the sample demonstrated good adherence. These findings are consistent with the progressive abandonment of the Mediterranean dietary pattern in favor of the Western diet reported in the literature [39,40]. In terms of specific eating habits, approximately 9% of the children reported not having breakfast daily, a slightly lower proportion than that estimated in 2023 (10.9%) [11]. Among those who regularly consume breakfast, nearly 70% reported eating snacks, biscuits, packaged pastries or other sweet products. The data regarding daily fruit and vegetable consumption highlight a concerning situation: about a third of the children reported not consuming fruit and/or vegetables every day, in line with the findings reported by the OKkio alla SALUTE report [11].

Moreover, 42.5% of the children reported regularly consuming fruit juices or sugar-sweetened carbonated beverages during snacks, and all the students stated that their snack consisted of sweet snacks, savory snacks, or both. Less than half of the children reported consuming milk or yogurt during their snack, while slightly more than half reported consuming fruit. Overall, these findings suggest that snacks frequently represent nutritionally unbalanced and hypercaloric meals. Consistently, data from the OKkio alla SALUTE report indicate that 66.9% of children consume a mid-morning snack that is considered to be excessively abundant and therefore nutritionally inappropriate [11].

With regard to hydration habits, less than half of the sample reported drinking only one bottle of water during the entire school day, and nearly one third reported drinking even less than one. Such habits may promote the consumption of sugar-sweetened and carbonated beverages, which have been shown to negatively affect both weight status and oral health [41]. Finally, some children reported consuming sweets or candies several times per day and visiting fast-food restaurants more than once per week.

Overall, these findings suggest that unhealthy dietary habits remain largely prevalent among 9–10-year-old children in Lombardy. Such behaviors may contribute to both excess body weight and the development of dental caries, particularly due to the high intake of simple sugars [12].

With regard to oral health status, two indicators were analyzed: the plaque index according to the O’Leary Plaque Index method and the epidemiological caries index, the DMFT/dmft index [26]. The mean plaque index observed in the sample was 80%, with limited variability among schools; this value is markedly higher than the reference threshold established by the World Health Organization, which is set at 30% [2].

The mean DMFT/dmft value recorded in the sample was 0.7 (SD = 1.26), with notable differences among the schools. Also in this case, the observed value exceeds the target of 0.5 established by the World Health Organization for 12-year-old children by 2020 [2]. Within the study sample, nearly half of the children had already experienced dental caries and approximately 10% had already lost at least one tooth due to the disease (9.9% primary teeth). Clinical examinations conducted during the educational sessions further revealed that 30% of the children presented DMFT ≥ 1. Overall, these findings suggest that the oral health status of fourth-grade children in Lombardy is far from optimal.

Several domestic oral hygiene practices were also investigated in order to assess their prevalence in the sample and to better understand the level of parental awareness. Nearly one quarter of the parents reported that their child brushes their teeth only once per day, either in the morning or in the evening. Thus, they are not complying with the recommendations of the Italian Ministry of Health. These guidelines recommend that children aged 6–12 years brush their teeth at least twice daily using toothpaste containing 1000 ppm of fluoride in order to maintain adequate oral health [42].

Encouragingly, the collected data showed that only 1.3% of the children do not use toothpaste during tooth brushing, whereas the majority report daily use. However, knowledge of toothpaste composition—particularly fluoride content—is also crucial. More than one quarter of the parents reported either not using fluoridated toothpaste for their children or being unaware of whether the toothpaste used contains fluoride.

Regarding the adequacy of oral hygiene tools for the child’s age, the majority of parents reported that their child uses a toothbrush and toothpaste specifically designed for children. Nevertheless, approximately 5% of the parents were not aware of these age-specific recommendations.

Taken together, these findings suggest that both oral hygiene practices and parental awareness and attention toward these practices could still be improved.

The adoption of appropriate health behaviors during childhood begins within the home environment and is strongly influenced by parents, particularly mothers. For this reason, parents should be adequately informed about how their own hygiene habits—and general lifestyle behaviors—may influence their children’s oral health and, consequently, their overall quality of life [43].

Another relevant finding concerns the mean age at the first dental visit, which in the present sample is 5.8 years (SD = 1.62), considerably later than what is recommended by the clinical guidelines, according to which the first dental visit should ideally occur within the first 12 months of life [42]. Although the majority of the parents reported regularly taking their children to the dentist, nearly 10% of the sample had never undergone a dental visit. Furthermore, almost 15% of the parents indicated “tooth pain” as the reason for the first dental visit, suggesting that dental care is often sought only when the disease has already progressed to a symptomatic and advanced stage.

In this context, the importance of early preventive interventions should be strongly emphasized.

The results of the regression model are consistent with the findings reported in the literature.

In particular, higher plaque index values were associated with a markedly higher predicted probability of DMFT/dmft ≥ 1. An elevated plaque index generally reflects poor, or even absent, oral hygiene practices, which is known to promote the development of dental caries [1].

When children had never experienced dental caries the predicted probability of DMFT/dmft ≥ 1 was substantially lower than when there was a previous history of cavities. This finding aligns with the widely accepted view that dental caries is a chronic and recurrent disease [1].

With regard to dietary habits, skipping breakfast was associated with a higher predicted probability of DMFT/dmft ≥ 1 compared with children who usually eat breakfast. This finding is in line with previous evidence suggesting that children who do not consume breakfast are more likely to consume less healthy and more palatable snacks (usually rich in simple sugars) during the morning. Furthermore, children who skip breakfast may also be less likely to brush their teeth in the morning. Both behaviors may help to explain the observed association with prevalent caries status [16].

Conversely, not consuming a mid-morning snack was associated with a lower predicted probability of DMFT/dmft≥ 1 compared with children who do have a snack at school. One possible explanation is that mid-morning snacks are typically consumed at school, where children generally do not have the opportunity to brush their teeth immediately afterward. Consequently, food residues may remain in the oral cavity for prolonged periods, potentially persisting until the end of the day, creating an environment that is conducive to the development of dental caries [1,8]. In the present sample, only about 20% of the children reported brushing their teeth more than twice per day, which may further contribute to the persistence of food residues and may be consistent with the observed association with prevalent caries status. Not consuming fruit every day was associated with a lower probability of prevalent caries status (DMFT/dmft ≥ 1) relative to daily fruit consumption. This finding remains controversial as the scientific community has not reached a consensus regarding the potential cariogenicity of fruit. Given the low sample size and the partly data-driven variable selection process, this result should not be interpreted as evidence that lower fruit intake is protective against caries. One possible explanation is that fruit is often consumed at times of the day when toothbrushing is not possible or not routinely practiced. According to these results, plaque index and previous history of caries may be considered as proximal markers of prevalent caries status.

The present study has several limitations. First, because of the cross-sectional design, no causal interpretation should be attributed to the observed associations, and the retained variables cannot be interpreted as predictors of future caries occurrence but only as variables that are associated with prevalent caries status at the time of examination. The model addresses prevalent caries status at the time of examination and does not provide information on the temporal relationship of exposures and outcome. Second, the participating schools were selected by convenience rather than through probability sampling. Therefore, the analyzed sample cannot be assumed to be representative of all school-aged children in Lombardy or Northern Italy. Furthermore, participant selection into the final analysis may have been affected by non-ignorable mechanisms, i.e., a lack of parental consent, falsified questionnaires concentrated in one school, or absence on the day of data collection. Although the analysis of the demographic characteristics of the children from Montello school does not suggest differences between the excluded and included children, the possibility of selection bias should be acknowledged. Unfortunately, its magnitude and direction could not be reliably evaluated because questionnaire data were not available for most of the excluded children and were considered to be unreliable for those with falsified questionnaires. Third, the outcome was dichotomized as DMFT/dmft 0 versus >=1. Although this choice improved interpretability and modeling feasibility, it discarded information on severity and combined primary and permanent dentition into a single endpoint. Fourth, the modeling strategy involved a partly data-driven reduction in candidate variables. As a consequence, formal inference after variable selection is not valid and could not be reported, which limits the extent to which the observed associations can be inferred beyond the analyzed sample. Although internal bootstrap validation was used and provided useful information on optimism, variable stability, discrimination, and calibration, no external validation dataset was available, and the model was developed in a relatively small sample. In addition, calibration was heterogeneous across schools, with wide confidence intervals, indicating that the developed model did not perform uniformly across clusters and that it tended to underestimate the observed prevalent caries status in some schools. Furthermore, the assessment of model stability across bootstrap samples showed that, although the retained factors were individually among the most frequently selected variables, the final model specification itself was less stable. Therefore, the retained variables should not be interpreted as the only plausible combination of factors associated with the observed prevalent caries status. Consequently, the internally validated performance reported here should be interpreted cautiously and regarded as exploratory. In particular, given the possibility of selection bias and the absence of external validation, further evaluation is needed to establish the model’s generalizability. Despite these limitations, this study provides a useful descriptive overview of dietary and oral hygiene behaviors in a school-based regional sample and identifies variables that may deserve further investigation in future studies.

## 5. Conclusions

The dietary habits and oral health conditions among 9–10-year-old children in Lombardy remain suboptimal. Given that both dental caries and obesity are largely preventable conditions, the implementation of initiatives aimed at increasing awareness among parents and children regarding healthy nutrition, appropriate home oral hygiene practices, and the importance of regular dental visits is essential. As children in this age group spend a substantial portion of their time at school, the school setting represents a particularly relevant environment for promoting such initiatives and encouraging their uptake. Moreover, plaque index and previous history of caries were associated with prevalent caries status in the analyzed sample and may warrant further investigation in future studies to assess their potential usefulness in the prevention and management of dental caries.

## Figures and Tables

**Table 1:** Number of children for schools, initial sample.

	Total (n = 497)
School, n (%)	
Monza Buonarroti	47 (9.5%)
Monza Manzoni	44 (8.9%)
Monza Citterio	38 (7.6%)
Desio Collegio Di Rosa	50 (10.1%)
Madone	30 (6.0%)
Merate Sartirana	33 (6.6%)
Villa D’Adda	39 (7.8%)
Mapello	39 (7.8%)
Bonate Sotto	44 (8.9%)
Merate Montello	63 (12.7%)
Brembate Sopra	70 (14.1%)

**Table 2:** Number of consents not given and falsified.

	Consent Not Given (n = 32)	Falsified (n = 50)	Total (n = 82)
School, n (%)			
Monza Buonarroti	5 (15.6%)	0 (0.0%)	5 (6.1%)
Monza Manzoni	1 (3.1%)	0 (0.0%)	1 (1.2%)
Monza Citterio	2 (6.3%)	0 (0.0%)	2 (2.4%)
Desio Collegio Di Rosa	2 (6.3%)	0 (0.0%)	2 (2.4%)
Madone	1 (3.1%)	0 (0.0%)	1 (1.2%)
Merate Sartirana	7 (21.9%)	0 (0.0%)	7 (8.5%)
Villa D’Adda	2 (6.3%)	0 (0.0%)	2 (2.4%)
Mapello	2 (6.3%)	0 (0.0%)	2 (2.4%)
Bonate Sotto	0 (0.0%)	0 (0.0%)	0 (0.0%)
Merate Montello	4 (12.5%)	50 (100.0%)	54 (65.9%)
Brembate Sopra	6 (18.8%)	0 (0.0%)	6 (7.3%)

**Table 3:** Number of absences from school and lost questionnaires.

	Absent from School (n = 11)	Lost Questionnaire (n = 96)	Total (n = 107)
School, n (%)			
Monza Buonarroti	0 (0.0%)	10 (10.4%)	10 (9.3%)
Monza Manzoni	2 (18.2%)	6 (6.3%)	8 (7.5%)
Monza Citterio	3 (27.3%)	11 (11.5%)	14 (13.1%)
Desio Collegio Di Rosa	3 (27.3%)	8 (8.3%)	11 (10.3%)
Madone	0 (0.0%)	5 (5.2%)	5 (4.7%)
Merate Sartirana	0 (0.0%)	5 (5.2%)	5 (4.7%)
Villa D’Adda	0 (0.0%)	3 (3.1%)	3 (2.8%)
Mapello	1 (9.1%)	9 (9.4%)	10 (9.3%)
Bonate Sotto	0 (0.0%)	13 (13.5%)	13 (12.1%)
Merate Montello	1 (9.1%)	7 (7.3%)	8 (7.5%)
Brembate Sopra	1 (9.1%)	19 (19.8%)	20 (18.7%)

**Table 4:** Number of children for school. Analyzed sample.

	Total (n = 307)
School, n (%)	
Monza Buonarroti	32 (10.4%)
Monza Manzoni	35 (11.4%)
Monza Citterio	22 (7.2%)
Desio Collegio Di Rosa	37 (12.1%)
Madone	24 (7.8%)
Merate Sartirana	21 (6.8%)
Villa D’Adda	34 (11.1%)
Mapello	27 (8.8%)
Bonate Sotto	31 (10.1%)
Brembate Sopra	44 (14.3%)

**Table 5:** Demographic characteristics.

	School										
	Monza Buonarroti (n = 32)	Monza Manzoni (n = 35)	Monza Citterio (n = 22)	Desio Collegio Di Rosa (n = 37)	Madone (n = 24)	Merate Sartirana (n = 21)	Villa D’Adda (n = 34)	Mapello (n = 27)	Bonate Sotto (n = 31)	Brembate Sopra (n = 44)	Total (n = 307)
Child age											
N	32	35	22	37	24	21	34	27	31	44	307
Mean (SD)	9.1 (0.25)	9.1 (0.28)	9.2 (0.39)	9.2 (0.37)	9.2 (0.64)	9.0 (0.22)	9.3 (0.45)	9.2 (0.42)	9.3 (0.44)	9.4 (0.50)	9.2 (0.42)
Median	9.0	9.0	9.0	9.0	9.0	9.0	9.0	9.0	9.0	9.0	9.0
Range	9.0, 10.0	9.0, 10.0	9.0, 10.0	9.0, 10.0	9.0, 12.0	9.0, 10.0	9.0, 10.0	9.0, 10.0	9.0, 10.0	9.0, 10.0	9.0, 12.0
Gender, n (%)											
Male	11 (34.4%)	22 (62.9%)	10 (45.5%)	22 (59.5%)	14 (58.3%)	11 (52.4%)	17 (50.0%)	12 (44.4%)	14 (45.2%)	22 (50.0%)	155 (50.5%)
Female	21 (65.6%)	13 (37.1%)	12 (54.5%)	15 (40.5%)	10 (41.7%)	10 (47.6%)	17 (50.0%)	15 (55.6%)	17 (54.8%)	22 (50.0%)	152 (49.5%)
Child nationality, n (%)											
Foreign	4 (13.8%)	2 (5.7%)	3 (13.6%)	0 (0.0%)	3 (14.3%)	0 (0.0%)	0 (0.0%)	0 (0.0%)	0 (0.0%)	0 (0.0%)	12 (4.0%)
Italian	25 (86.2%)	33 (94.3%)	19 (86.4%)	37 (100.0%)	18 (85.7%)	20 (100.0%)	34 (100.0%)	27 (100.0%)	31 (100.0%)	44 (100.0%)	288 (96.0%)
Missing	3	0	0	0	3	1	0	0	0	0	7
Parents’ nationality, n (%)											
Both foreign	9 (29.0%)	2 (5.9%)	8 (40.0%)	0 (0.0%)	8 (33.3%)	0 (0.0%)	1 (2.9%)	6 (22.2%)	2 (6.5%)	1 (2.3%)	37 (12.2%)
Both Italian	19 (61.3%)	31 (91.2%)	11 (55.0%)	36 (97.3%)	15 (62.5%)	19 (90.5%)	32 (94.1%)	21 (77.8%)	24 (77.4%)	38 (86.4%)	246 (81.2%)
Only one Italian parent	3 (9.7%)	1 (2.9%)	1 (5.0%)	1 (2.7%)	1 (4.2%)	2 (9.5%)	1 (2.9%)	0 (0.0%)	5 (16.1%)	5 (11.4%)	20 (6.6%)
Missing	1	1	2	0	0	0	0	0	0	0	4
Parents’ education, n (%)											
Middle school or less	4 (13.3%)	7 (20.0%)	3 (13.6%)	2 (5.6%)	11 (45.8%)	2 (9.5%)	3 (8.8%)	8 (29.6%)	7 (22.6%)	11 (25.0%)	58 (19.1%)
High school	10 (33.3%)	12 (34.3%)	11 (50.0%)	19 (52.8%)	12 (50.0%)	10 (47.6%)	19 (55.9%)	15 (55.6%)	19 (61.3%)	23 (52.3%)	150 (49.3%)
Degree	16 (53.3%)	16 (45.7%)	8 (36.4%)	15 (41.7%)	1 (4.2%)	9 (42.9%)	12 (35.3%)	4 (14.8%)	5 (16.1%)	10 (22.7%)	96 (31.6%)
Missing	2	0	0	1	0	0	0	0	0	0	3
Classification of weight status according to Cole class											
N	32	35	22	37	24	21	34	27	31	44	307
Underweight	3 (9.4%)	1 (2.9%)	0 (0.0%)	2 (5.4%)	0 (0.0%)	1 (4.8%)	2 (5.9%)	1 (3.7%)	0 (0.0%)	2 (4.5%)	12 (3.9%)
Normal weight	20 (62.5%)	22 (62.9%)	13 (59.1%)	30 (81.1%)	15 (62.5%)	19 (90.5%)	26 (76.5%)	19 (70.4%)	25 (80.6%)	34 (77.3%)	223 (72.6%)
Overweight	7 (21.9%)	11 (31.4%)	8 (36.4%)	5 (13.5%)	8 (33.3%)	1 (4.8%)	5 (14.7%)	6 (22.2%)	4 (12.9%)	7 (15.9%)	62 (21.2%)
Obese	2 (6.3%)	1 (2.9%)	1 (4.5%)	0 (0.0%)	1 (4.2%)	0 (0.0%)	1 (2.9%)	1 (3.7%)	2 (6.5%)	1 (2.3%)	10 (3.3%)
Mediterranean diet adherence index (parents)											
N	31	35	22	37	24	21	34	27	31	44	306
Mean (SD)	4.9 (2.49)	5.5 (2.36)	5.4 (2.66)	4.8 (2.50)	4.3 (3.09)	5.6 (2.64)	5.2 (2.41)	4.7 (2.19)	5.1 (2.10)	4.7 (2.49)	5.0 (2.47)
Median	5.0	5.0	6.0	5.0	5.0	6.0	5.0	4.0	5.0	5.0	5.0
Range	0.0, 11.0	0.0, 10.0	0.0, 10.0	1.0, 10.0	−1.0, 11.0	1.0, 9.0	1.0, 10.0	1.0, 9.0	1.0, 8.0	−1.0, 11.0	−1.0, 11.0
Mediterranean diet adherence index (child)											
N	32	35	22	37	24	21	34	27	31	44	307
Mean (SD)	4.5 (2.68)	5.6 (2.77)	5.3 (2.10)	5.2 (2.47)	5.5 (2.70)	5.7 (2.92)	6.1 (2.56)	6.5 (2.19)	5.6 (2.43)	6.1 (2.71)	5.6 (2.59)
Median	4.0	6.0	6.0	6.0	6.0	7.0	6.5	7.0	6.0	6.5	6.0
Range	0.0, 9.0	0.0, 11.0	0.0, 9.0	1.0, 10.0	−1.0, 11.0	0.0, 10.0	1.0, 11.0	2.0, 10.0	1.0, 11.0	0.0, 10.0	−1.0, 11.0
Mediterranean diet adherence index in class, n (%)											
Low adherence	13 (40.6%)	9 (25.7%)	5 (22.7%)	11 (29.7%)	5 (20.8%)	5 (23.8%)	6 (17.6%)	2 (7.4%)	6 (19.4%)	9 (20.5%)	71 (23.1%)
Moderate adherence	12 (37.5%)	18 (51.4%)	14 (63.6%)	19 (51.4%)	15 (62.5%)	11 (52.4%)	18 (52.9%)	16 (59.3%)	19 (61.3%)	21 (47.7%)	163 (53.1%)
High adherence	7 (21.9%)	8 (22.9%)	3 (13.6%)	7 (18.9%)	4 (16.7%)	5 (23.8%)	10 (29.4%)	9 (33.3%)	6 (19.4%)	14 (31.8%)	73 (23.8%)

**Table 6:** Snack questionnaire.

	School										
	Monza Buonarroti (n = 32)	Monza Manzoni (n = 35)	Monza Citterio (n = 22)	Desio Collegio Di Rosa (n = 37)	Madone (n = 24)	Merate Sartirana (n = 21)	Villa D’Adda (n = 34)	Mapello (n = 27)	Bonate Sotto (n = 31)	Brembate Sopra (n = 44)	Total (n = 307)
Do you eat breakfast on the days you go to school? Child, n (%)											
No	3 (9.4%)	2 (5.7%)	2 (9.1%)	2 (5.4%)	3 (12.5%)	2 (9.5%)	1 (2.9%)	3 (11.1%)	5 (16.1%)	3 (6.8%)	26 (8.5%)
Yes	29 (90.6%)	33 (94.3%)	20 (90.9%)	35 (94.6%)	21 (87.5%)	19 (90.5%)	33 (97.1%)	24 (88.9%)	26 (83.9%)	41 (93.2%)	281 (91.5%)
Do you eat breakfast on the weekend? Child, n (%)											
No	3 (9.4%)	1 (2.9%)	1 (4.5%)	1 (2.7%)	3 (12.5%)	0 (0.0%)	0 (0.0%)	1 (3.7%)	5 (16.1%)	1 (2.3%)	16 (5.2%)
Yes	29 (90.6%)	34 (97.1%)	21 (95.5%)	36 (97.3%)	21 (87.5%)	21 (100.0%)	34 (100.0%)	26 (96.3%)	26 (83.9%)	43 (97.7%)	291 (94.8%)
Do you have a mid-morning snack at school? Child, n (%)											
No	2 (6.3%)	1 (2.9%)	1 (4.8%)	32 (86.5%)	1 (4.2%)	0 (0.0%)	0 (0.0%)	1 (3.7%)	2 (6.5%)	1 (2.3%)	41 (13.4%)
Yes	30 (93.8%)	34 (97.1%)	20 (95.2%)	5 (13.5%)	23 (95.8%)	21 (100.0%)	34 (100.0%)	26 (96.3%)	29 (93.5%)	43 (97.7%)	265 (86.6%)
Missing	0	0	1	0	0	0	0	0	0	0	1
Do you have a mid-morning snack on weekends? Child, n (%)											
No	20 (62.5%)	22 (62.9%)	16 (76.2%)	28 (75.7%)	16 (66.7%)	18 (85.7%)	26 (76.5%)	20 (74.1%)	27 (87.1%)	34 (77.3%)	227 (74.2%)
Yes	12 (37.5%)	13 (37.1%)	5 (23.8%)	9 (24.3%)	8 (33.3%)	3 (14.3%)	8 (23.5%)	7 (25.9%)	4 (12.9%)	10 (22.7%)	79 (25.8%)
Missing	0	0	1	0	0	0	0	0	0	0	1
What do you usually eat for snack? Child, n (%)											
Sweet foods	5 (20.8%)	16 (45.7%)	9 (45.0%)	8 (21.6%)	5 (21.7%)	4 (21.1%)	8 (25.0%)	7 (29.2%)	3 (11.1%)	10 (25.6%)	75 (26.8%)
Salty foods	11 (45.8%)	4 (11.4%)	2 (10.0%)	10 (27.0%)	9 (39.1%)	5 (26.3%)	13 (40.6%)	9 (37.5%)	8 (29.6%)	16 (41.0%)	87 (31.1%)
Both	8 (33.3%)	15 (42.9%)	9 (45.0%)	19 (51.4%)	9 (39.1%)	10 (52.6%)	11 (34.4%)	8 (33.3%)	16 (59.3%)	13 (33.3%)	118 (42.1%)
Missing	8	0	2	0	1	2	2	3	4	5	27
What do you usually drink at snack time? Child, n (%)											
Juice	13 (40.6%)	21 (60.0%)	6 (27.3%)	14 (38.9%)	9 (37.5%)	9 (42.9%)	5 (15.2%)	15 (55.6%)	15 (48.4%)	14 (33.3%)	121 (39.9%)
Milk	1 (3.1%)	2 (5.7%)	0 (0.0%)	4 (11.1%)	1 (4.2%)	0 (0.0%)	4 (12.1%)	1 (3.7%)	1 (3.2%)	1 (2.4%)	15 (5.0%)
Cola/Fanta/Sprite	1 (3.1%)	0 (0.0%)	0 (0.0%)	1 (2.8%)	2 (8.3%)	0 (0.0%)	1 (3.0%)	0 (0.0%)	1 (3.2%)	2 (4.8%)	8 (2.6%)
Other beverages	17 (53.1%)	12 (34.3%)	16 (72.7%)	17 (47.2%)	12 (50.0%)	12 (57.1%)	23 (69.7%)	11 (40.7%)	14 (45.2%)	25 (59.5%)	159 (52.5%)
Missing	0	0	0	1	0	0	1	0	0	2	4
How much water do you drink at school? Child, n (%)											
More than a bottle	8 (25.0%)	6 (17.1%)	2 (9.1%)	6 (16.2%)	6 (25.0%)	3 (15.0%)	5 (14.7%)	7 (25.9%)	5 (16.1%)	10 (23.8%)	58 (19.1%)
One bottle	13 (40.6%)	15 (42.9%)	13 (59.1%)	12 (32.4%)	10 (41.7%)	6 (30.0%)	21 (61.8%)	12 (44.4%)	13 (41.9%)	21 (50.0%)	136 (44.7%)
Less than a bottle	11 (34.4%)	14 (40.0%)	7 (31.8%)	19 (51.4%)	8 (33.3%)	11 (55.0%)	8 (23.5%)	8 (29.6%)	13 (41.9%)	11 (26.2%)	110 (36.2%)
Missing	0	0	0	0	0	1	0	0	0	2	3
Do you eat fruit as a snack? Child, n (%)											
No	14 (43.8%)	9 (25.7%)	11 (50.0%)	13 (35.1%)	8 (36.4%)	6 (28.6%)	15 (45.5%)	10 (37.0%)	14 (45.2%)	14 (32.6%)	114 (37.6%)
Yes	18 (56.3%)	26 (74.3%)	11 (50.0%)	24 (64.9%)	14 (63.6%)	15 (71.4%)	18 (54.5%)	17 (63.0%)	17 (54.8%)	29 (67.4%)	189 (62.4%)
Missing	0	0	0	0	2	0	1	0	0	1	4
Do you eat yogurt or drink milk as a snack? Child, n (%)											
No	17 (53.1%)	20 (57.1%)	12 (54.5%)	24 (64.9%)	11 (45.8%)	17 (81.0%)	22 (64.7%)	14 (53.8%)	20 (64.5%)	23 (53.5%)	180 (59.0%)
Yes	15 (46.9%)	15 (42.9%)	10 (45.5%)	13 (35.1%)	13 (54.2%)	4 (19.0%)	12 (35.3%)	12 (46.2%)	11 (35.5%)	20 (46.5%)	125 (41.0%)
Missing	0	0	0	0	0	0	0	1	0	1	2
Do you bring snacks from home to school? Child, n (%)											
No	2 (6.3%)	1 (2.9%)	0 (0.0%)	28 (75.7%)	2 (8.7%)	0 (0.0%)	0 (0.0%)	1 (3.7%)	2 (6.5%)	2 (4.5%)	38 (12.4%)
Yes	30 (93.8%)	34 (97.1%)	22 (100.0%)	9 (24.3%)	21 (91.3%)	21 (100.0%)	34 (100.0%)	26 (96.3%)	29 (93.5%)	42 (95.5%)	268 (87.6%)
Missing	0	0	0	0	1	0	0	0	0	0	1
If you bring it from home, who makes it? Child, n (%)											
Child	16 (51.6%)	14 (41.2%)	12 (54.5%)	3 (33.3%)	10 (43.5%)	10 (47.6%)	20 (58.8%)	14 (51.9%)	18 (62.1%)	33 (75.0%)	150 (54.7%)
Parents	15 (48.4%)	18 (52.9%)	10 (45.5%)	5 (55.6%)	12 (52.2%)	11 (52.4%)	14 (41.2%)	13 (48.1%)	11 (37.9%)	9 (20.5%)	118 (43.1%)
Brothers/sisters	0 (0.0%)	0 (0.0%)	0 (0.0%)	1 (11.1%)	0 (0.0%)	0 (0.0%)	0 (0.0%)	0 (0.0%)	0 (0.0%)	1 (2.3%)	2 (0.7%)
Grandparents	0 (0.0%)	2 (5.9%)	0 (0.0%)	0 (0.0%)	1 (4.3%)	0 (0.0%)	0 (0.0%)	0 (0.0%)	0 (0.0%)	1 (2.3%)	4 (1.5%)
Missing	1	1	0	28	1	0	0	0	2	0	33
Do you snack in the afternoon after school? Child, n (%)											
No	6 (18.8%)	3 (8.6%)	5 (22.7%)	1 (2.7%)	3 (12.5%)	0 (0.0%)	1 (2.9%)	1 (3.7%)	2 (6.5%)	2 (4.5%)	24 (7.8%)
Yes	26 (81.3%)	32 (91.4%)	17 (77.3%)	36 (97.3%)	21 (87.5%)	21 (100.0%)	33 (97.1%)	26 (96.3%)	29 (93.5%)	42 (95.5%)	283 (92.2%)
Did you take nutritional education classes in the current or previous year? Child, n (%)											
No	6 (18.8%)	2 (5.7%)	5 (22.7%)	23 (62.2%)	6 (25.0%)	3 (14.3%)	8 (23.5%)	10 (37.0%)	21 (67.7%)	3 (6.8%)	87 (28.3%)
Yes	26 (81.3%)	33 (94.3%)	17 (77.3%)	14 (37.8%)	18 (75.0%)	18 (85.7%)	26 (76.5%)	17 (63.0%)	10 (32.3%)	41 (93.2%)	220 (71.7%)
How many times a day do you brush your teeth? Child, n (%)											
Never	0 (0.0%)	0 (0.0%)	0 (0.0%)	0 (0.0%)	1 (4.2%)	0 (0.0%)	0 (0.0%)	0 (0.0%)	0 (0.0%)	0 (0.0%)	1 (0.3%)
Once	5 (15.6%)	7 (20.0%)	3 (13.6%)	5 (13.5%)	6 (25.0%)	3 (14.3%)	2 (5.9%)	7 (25.9%)	2 (6.5%)	8 (18.2%)	48 (15.6%)
Twice	22 (68.8%)	23 (65.7%)	12 (54.5%)	22 (59.5%)	14 (58.3%)	14 (66.7%)	25 (73.5%)	17 (63.0%)	18 (58.1%)	27 (61.4%)	194 (63.2%)
More than twice	5 (15.6%)	5 (14.3%)	7 (31.8%)	10 (27.0%)	3 (12.5%)	4 (19.0%)	7 (20.6%)	3 (11.1%)	11 (35.5%)	9 (20.5%)	64 (20.8%)
Do you brush your teeth after breakfast before going to school? Child, n (%)											
Always	26 (81.3%)	30 (85.7%)	16 (72.7%)	29 (78.4%)	14 (58.3%)	16 (76.2%)	26 (76.5%)	21 (77.8%)	22 (71.0%)	29 (65.9%)	229 (74.6%)
Frequently	2 (6.3%)	3 (8.6%)	3 (13.6%)	5 (13.5%)	7 (29.2%)	2 (9.5%)	6 (17.6%)	3 (11.1%)	1 (3.2%)	5 (11.4%)	37 (12.1%)
A little	1 (3.1%)	0 (0.0%)	1 (4.5%)	2 (5.4%)	1 (4.2%)	2 (9.5%)	0 (0.0%)	1 (3.7%)	7 (22.6%)	4 (9.1%)	19 (6.2%)
Never	3 (9.4%)	2 (5.7%)	2 (9.1%)	1 (2.7%)	2 (8.3%)	1 (4.8%)	2 (5.9%)	2 (7.4%)	1 (3.2%)	6 (13.6%)	22 (7.2%)
Do you brush your teeth after breakfast on Saturdays and Sundays? Child, n (%)											
Always	17 (53.1%)	22 (62.9%)	9 (40.9%)	24 (64.9%)	13 (54.2%)	12 (57.1%)	21 (61.8%)	19 (70.4%)	18 (60.0%)	20 (45.5%)	175 (57.2%)
Frequently	3 (9.4%)	4 (11.4%)	5 (22.7%)	7 (18.9%)	7 (29.2%)	5 (23.8%)	7 (20.6%)	3 (11.1%)	5 (16.7%)	8 (18.2%)	54 (17.6%)
A little	7 (21.9%)	5 (14.3%)	3 (13.6%)	5 (13.5%)	3 (12.5%)	3 (14.3%)	4 (11.8%)	3 (11.1%)	5 (16.7%)	9 (20.5%)	47 (15.4%)
Never	5 (15.6%)	4 (11.4%)	5 (22.7%)	1 (2.7%)	1 (4.2%)	1 (4.8%)	2 (5.9%)	2 (7.4%)	2 (6.7%)	7 (15.9%)	30 (9.8%)
Missing	0	0	0	0	0	0	0	0	1	0	1
Do you brush your teeth before going to bed? Child, n (%)											
Always	22 (68.8%)	25 (71.4%)	11 (50.0%)	26 (70.3%)	13 (54.2%)	17 (81.0%)	26 (76.5%)	20 (74.1%)	23 (74.2%)	30 (68.2%)	213 (69.4%)
Frequently	5 (15.6%)	6 (17.1%)	7 (31.8%)	5 (13.5%)	8 (33.3%)	2 (9.5%)	6 (17.6%)	2 (7.4%)	3 (9.7%)	11 (25.0%)	55 (17.9%)
A little	2 (6.3%)	1 (2.9%)	2 (9.1%)	4 (10.8%)	2 (8.3%)	2 (9.5%)	2 (5.9%)	2 (7.4%)	3 (9.7%)	1 (2.3%)	21 (6.8%)
Never	3 (9.4%)	3 (8.6%)	2 (9.1%)	2 (5.4%)	1 (4.2%)	0 (0.0%)	0 (0.0%)	3 (11.1%)	2 (6.5%)	2 (4.5%)	18 (5.9%)

**Table 7:** KIDMED items.

	School										
	Monza Buonarroti (n = 32)	Monza Manzoni (n = 35)	Monza Citterio (n = 22)	Desio Collegio Di Rosa (n = 37)	Madone (n = 24)	Merate Sartirana (n = 21)	Villa D’Adda (n = 34)	Mapello (n = 27)	Bonate Sotto (n = 31)	Brembate Sopra (n = 44)	Total (n = 307)
Do you consume a fruit every day? Child, n (%)											
No	9 (28.1%)	8 (22.9%)	5 (22.7%)	16 (43.2%)	7 (29.2%)	7 (33.3%)	8 (23.5%)	8 (29.6%)	6 (19.4%)	10 (22.7%)	84 (27.4%)
Yes	23 (71.9%)	27 (77.1%)	17 (77.3%)	21 (56.8%)	17 (70.8%)	14 (66.7%)	26 (76.5%)	19 (70.4%)	25 (80.6%)	34 (77.3%)	223 (72.6%)
Do you eat a second fruit every day? Child, n (%)											
No	19 (59.4%)	21 (60.0%)	12 (54.5%)	24 (64.9%)	15 (62.5%)	14 (66.7%)	19 (55.9%)	12 (44.4%)	21 (67.7%)	20 (45.5%)	177 (57.7%)
Yes	13 (40.6%)	14 (40.0%)	10 (45.5%)	13 (35.1%)	9 (37.5%)	7 (33.3%)	15 (44.1%)	15 (55.6%)	10 (32.3%)	24 (54.5%)	130 (42.3%)
Do you eat raw or cooked vegetables regularly 1 time a day? Child, n (%)											
No	13 (40.6%)	11 (31.4%)	7 (31.8%)	12 (32.4%)	10 (41.7%)	5 (23.8%)	5 (14.7%)	4 (14.8%)	10 (32.3%)	18 (40.9%)	95 (30.9%)
Yes	19 (59.4%)	24 (68.6%)	15 (68.2%)	25 (67.6%)	14 (58.3%)	16 (76.2%)	29 (85.3%)	23 (85.2%)	21 (67.7%)	26 (59.1%)	212 (69.1%)
Do you eat raw or cooked vegetables regularly more than 1 time a day? Child, n (%)											
No	26 (81.3%)	22 (62.9%)	12 (54.5%)	22 (59.5%)	19 (79.2%)	13 (61.9%)	18 (52.9%)	16 (59.3%)	19 (61.3%)	30 (68.2%)	197 (64.2%)
Yes	6 (18.8%)	13 (37.1%)	10 (45.5%)	15 (40.5%)	5 (20.8%)	8 (38.1%)	16 (47.1%)	11 (40.7%)	12 (38.7%)	14 (31.8%)	110 (35.8%)
Do you consume fish (at least 2–3 times a week)? Child, n (%)											
No	24 (75.0%)	19 (54.3%)	16 (72.7%)	24 (64.9%)	11 (45.8%)	14 (66.7%)	23 (67.6%)	13 (48.1%)	20 (64.5%)	21 (47.7%)	185 (60.3%)
Yes	8 (25.0%)	16 (45.7%)	6 (27.3%)	13 (35.1%)	13 (54.2%)	7 (33.3%)	11 (32.4%)	14 (51.9%)	11 (35.5%)	23 (52.3%)	122 (39.7%)
Do you go more than 1 time a week to fast food? Child, n (%)											
No	25 (78.1%)	29 (82.9%)	20 (90.9%)	34 (91.9%)	18 (75.0%)	20 (95.2%)	33 (97.1%)	24 (88.9%)	27 (87.1%)	36 (81.8%)	266 (86.6%)
Yes	7 (21.9%)	6 (17.1%)	2 (9.1%)	3 (8.1%)	6 (25.0%)	1 (4.8%)	1 (2.9%)	3 (11.1%)	4 (12.9%)	8 (18.2%)	41 (13.4%)
Do you eat legumes more than 1 time a week? Child, n (%)											
No	17 (53.1%)	13 (37.1%)	7 (31.8%)	13 (35.1%)	8 (33.3%)	7 (33.3%)	13 (38.2%)	11 (40.7%)	14 (45.2%)	24 (54.5%)	127 (41.4%)
Yes	15 (46.9%)	22 (62.9%)	15 (68.2%)	24 (64.9%)	16 (66.7%)	14 (66.7%)	21 (61.8%)	16 (59.3%)	17 (54.8%)	20 (45.5%)	180 (58.6%)
Do you consume pasta or rice (5 or more times a week)? Child, n (%)											
No	9 (28.1%)	4 (11.4%)	8 (36.4%)	10 (27.0%)	3 (12.5%)	4 (19.0%)	12 (35.3%)	5 (18.5%)	5 (16.1%)	11 (25.0%)	71 (23.1%)
Yes	23 (71.9%)	31 (88.6%)	14 (63.6%)	27 (73.0%)	21 (87.5%)	17 (81.0%)	22 (64.7%)	22 (81.5%)	26 (83.9%)	33 (75.0%)	236 (76.9%)
For breakfast, do you eat cereal, bread or rusk? Child, n (%)											
No	15 (46.9%)	16 (45.7%)	8 (36.4%)	21 (56.8%)	11 (45.8%)	12 (57.1%)	14 (41.2%)	10 (37.0%)	14 (45.2%)	14 (31.8%)	135 (44.0%)
Yes	17 (53.1%)	19 (54.3%)	14 (63.6%)	16 (43.2%)	13 (54.2%)	9 (42.9%)	20 (58.8%)	17 (63.0%)	17 (54.8%)	30 (68.2%)	172 (56.0%)
Do you consume dried fruits (at least 2–3 times a week)? Child, n (%)											
No	19 (59.4%)	18 (51.4%)	13 (59.1%)	24 (64.9%)	11 (45.8%)	12 (57.1%)	24 (70.6%)	14 (51.9%)	19 (61.3%)	20 (45.5%)	174 (56.7%)
Yes	13 (40.6%)	17 (48.6%)	9 (40.9%)	13 (35.1%)	13 (54.2%)	9 (42.9%)	10 (29.4%)	13 (48.1%)	12 (38.7%)	24 (54.5%)	133 (43.3%)
For seasoning, do you use olive oil at home? Child, n (%)											
No	6 (18.8%)	6 (17.1%)	5 (22.7%)	4 (10.8%)	3 (12.5%)	3 (14.3%)	3 (8.8%)	5 (18.5%)	7 (22.6%)	5 (11.4%)	47 (15.3%)
Yes	26 (81.3%)	29 (82.9%)	17 (77.3%)	33 (89.2%)	21 (87.5%)	18 (85.7%)	31 (91.2%)	22 (81.5%)	24 (77.4%)	39 (88.6%)	260 (84.7%)
Do you usually eat breakfast? Child, n (%)											
No	3 (9.4%)	4 (11.4%)	3 (13.6%)	3 (8.1%)	3 (12.5%)	1 (4.8%)	1 (2.9%)	3 (11.1%)	4 (12.9%)	3 (6.8%)	28 (9.1%)
Yes	29 (90.6%)	31 (88.6%)	19 (86.4%)	34 (91.9%)	21 (87.5%)	20 (95.2%)	33 (97.1%)	24 (88.9%)	27 (87.1%)	41 (93.2%)	279 (90.9%)
Do you eat milk or dairy products for breakfast? Child, n (%)											
No	15 (46.9%)	14 (40.0%)	10 (45.5%)	13 (35.1%)	6 (25.0%)	9 (42.9%)	13 (38.2%)	8 (29.6%)	14 (45.2%)	17 (38.6%)	119 (38.8%)
Yes	17 (53.1%)	21 (60.0%)	12 (54.5%)	24 (64.9%)	18 (75.0%)	12 (57.1%)	21 (61.8%)	19 (70.4%)	17 (54.8%)	27 (61.4%)	188 (61.2%)
For breakfast, do you eat snacks or cookies? Child, n (%)											
No	8 (25.0%)	6 (17.1%)	4 (18.2%)	10 (27.0%)	7 (29.2%)	9 (42.9%)	15 (44.1%)	8 (29.6%)	9 (29.0%)	15 (34.1%)	91 (29.6%)
Yes	24 (75.0%)	29 (82.9%)	18 (81.8%)	27 (73.0%)	17 (70.8%)	12 (57.1%)	19 (55.9%)	19 (70.4%)	22 (71.0%)	29 (65.9%)	216 (70.4%)
Do you consume 2 yogurts and/or cheese daily? Child, n (%)											
No	20 (62.5%)	22 (62.9%)	13 (59.1%)	24 (64.9%)	15 (62.5%)	13 (61.9%)	18 (52.9%)	13 (48.1%)	12 (38.7%)	23 (52.3%)	173 (56.4%)
Yes	12 (37.5%)	13 (37.1%)	9 (40.9%)	13 (35.1%)	9 (37.5%)	8 (38.1%)	16 (47.1%)	14 (51.9%)	19 (61.3%)	21 (47.7%)	134 (43.6%)
Do you consume sweets and candy several times each day? Child, n (%)											
No	19 (59.4%)	25 (71.4%)	14 (63.6%)	26 (70.3%)	17 (70.8%)	14 (66.7%)	25 (73.5%)	20 (74.1%)	23 (74.2%)	34 (77.3%)	217 (70.7%)
Yes	13 (40.6%)	10 (28.6%)	8 (36.4%)	11 (29.7%)	7 (29.2%)	7 (33.3%)	9 (26.5%)	7 (25.9%)	8 (25.8%)	10 (22.7%)	90 (29.3%)

**Table 8:** Descriptive analysis of oral hygiene variables.

	School										
	Monza Buonarroti (n = 32)	Monza Manzoni (n = 35)	Monza Citterio (n = 22)	Desio Collegio Di Rosa (n = 37)	Madone (n = 24)	Merate Sartirana (n = 21)	Villa D’Adda (n = 34)	Mapello (n = 27)	Bonate Sotto (n = 31)	Brembate Sopra (n = 44)	Total (n = 307)
Age of first dental visit (years). Parents											
N (missing)	23 (9)	32 (3)	18 (4)	34 (3)	23 (1)	18 (3)	31 (3)	23 (4)	27 (4)	34 (10)	263 (44)
Mean (SD)	5.2 (1.78)	6.0 (1.51)	5.9 (1.70)	5.4 (1.43)	6.4 (1.41)	5.1 (1.26)	6.0 (1.80)	5.7 (1.63)	5.8 (1.48)	5.9 (1.82)	5.8 (1.62)
Does the child brush his teeth? Parents, n (%)											
Yes, every day	29 (96.7%)	34 (97.1%)	21 (95.5%)	37 (100.0%)	21 (87.5%)	20 (95.2%)	34 (100.0%)	24 (88.9%)	29 (93.5%)	42 (97.7%)	291 (95.7%)
Yes, some days	1 (3.3%)	1 (2.9%)	0 (0.0%)	0 (0.0%)	2 (8.3%)	0 (0.0%)	0 (0.0%)	2 (7.4%)	2 (6.5%)	1 (2.3%)	9 (3.0%)
Yes, few days	0 (0.0%)	0 (0.0%)	1 (4.5%)	0 (0.0%)	1 (4.2%)	1 (4.8%)	0 (0.0%)	1 (3.7%)	0 (0.0%)	0 (0.0%)	4 (1.3%)
Missing	2	0	0	0	0	0	0	0	0	1	3
The child brushes teeth aided by whom? Parents, n (%)											
On his/her own	26 (86.7%)	26 (74.3%)	20 (90.9%)	31 (83.8%)	21 (87.5%)	20 (95.2%)	31 (91.2%)	25 (96.2%)	27 (87.1%)	39 (88.6%)	266 (87.5%)
Helped by parents	0 (0.0%)	0 (0.0%)	1 (4.5%)	0 (0.0%)	0 (0.0%)	0 (0.0%)	2 (5.9%)	0 (0.0%)	0 (0.0%)	0 (0.0%)	3 (1.0%)
Both	4 (13.3%)	6 (17.1%)	1 (4.5%)	6 (16.2%)	3 (12.5%)	1 (4.8%)	1 (2.9%)	1 (3.8%)	4 (12.9%)	5 (11.4%)	32 (10.5%)
Don’t know	0 (0.0%)	3 (8.6%)	0 (0.0%)	0 (0.0%)	0 (0.0%)	0 (0.0%)	0 (0.0%)	0 (0.0%)	0 (0.0%)	0 (0.0%)	3 (1.0%)
Missing	2	0	0	0	0	0	0	1	0	0	3
How often does the child brush his teeth? Parents, n (%)											
In the morning	3 (10.0%)	5 (14.3%)	8 (36.4%)	3 (8.1%)	6 (25.0%)	1 (4.8%)	4 (11.8%)	3 (11.1%)	4 (12.9%)	3 (6.8%)	40 (13.1%)
In the evening	2 (6.7%)	3 (8.6%)	2 (9.1%)	0 (0.0%)	5 (20.8%)	2 (9.5%)	4 (11.8%)	1 (3.7%)	4 (12.9%)	7 (15.9%)	30 (9.8%)
Both	25 (83.3%)	27 (77.1%)	12 (54.5%)	34 (91.9%)	13 (54.2%)	18 (85.7%)	26 (76.5%)	23 (85.2%)	23 (74.2%)	34 (77.3%)	235 (77.0%)
Missing	2	0	0	0	0	0	0	0	0	0	2
What kind of toothbrush does the child use? Parents, n (%)											
Manual	15 (50.0%)	16 (45.7%)	12 (54.5%)	16 (43.2%)	12 (50.0%)	9 (42.9%)	14 (41.2%)	12 (44.4%)	9 (29.0%)	14 (31.8%)	129 (42.3%)
Electric	10 (33.3%)	16 (45.7%)	6 (27.3%)	18 (48.6%)	9 (37.5%)	11 (52.4%)	14 (41.2%)	12 (44.4%)	16 (51.6%)	25 (56.8%)	137 (44.9%)
Both	5 (16.7%)	3 (8.6%)	4 (18.2%)	3 (8.1%)	3 (12.5%)	1 (4.8%)	6 (17.6%)	3 (11.1%)	6 (19.4%)	5 (11.4%)	39 (12.8%)
Missing	2	0	0	0	0	0	0	0	0	0	2
Is it a baby toothbrush? Parents, n (%)											
No	3 (10.0%)	3 (8.6%)	3 (13.6%)	2 (5.4%)	0 (0.0%)	1 (4.8%)	2 (5.9%)	2 (7.4%)	1 (3.2%)	2 (4.5%)	19 (6.2%)
Yes	27 (90.0%)	32 (91.4%)	16 (72.7%)	32 (86.5%)	22 (91.7%)	19 (90.5%)	31 (91.2%)	21 (77.8%)	27 (87.1%)	39 (88.6%)	266 (87.2%)
Don’t know	0 (0.0%)	0 (0.0%)	3 (13.6%)	3 (8.1%)	2 (8.3%)	1 (4.8%)	1 (2.9%)	4 (14.8%)	3 (9.7%)	3 (6.8%)	20 (6.6%)
Missing	2	0	0	0	0	0	0	0	0	0	2
Is toothpaste used during brushing? Parents, n (%)											
Often	30 (100.0%)	35 (100.0%)	20 (90.9%)	35 (94.6%)	23 (95.8%)	21 (100.0%)	34 (100.0%)	25 (92.6%)	30 (96.8%)	43 (100.0%)	296 (97.4%)
Sometimes	0 (0.0%)	0 (0.0%)	1 (4.5%)	1 (2.7%)	1 (4.2%)	0 (0.0%)	0 (0.0%)	1 (3.7%)	0 (0.0%)	0 (0.0%)	4 (1.3%)
Never	0 (0.0%)	0 (0.0%)	1 (4.5%)	1 (2.7%)	0 (0.0%)	0 (0.0%)	0 (0.0%)	1 (3.7%)	1 (3.2%)	0 (0.0%)	4 (1.3%)
Missing	2	0	0	0	0	0	0	0	0	1	3
Does the toothpaste used contain fluoride? Parents, n (%)											
No	2 (6.7%)	3 (8.6%)	3 (13.6%)	4 (11.4%)	5 (20.8%)	4 (19.0%)	2 (5.9%)	2 (8.0%)	4 (12.9%)	3 (7.0%)	32 (10.7%)
Yes	21 (70.0%)	27 (77.1%)	10 (45.5%)	29 (82.9%)	16 (66.7%)	14 (66.7%)	30 (88.2%)	18 (72.0%)	21 (67.7%)	30 (69.8%)	216 (72.0%)
Don’t know	7 (23.3%)	5 (14.3%)	9 (40.9%)	2 (5.7%)	3 (12.5%)	3 (14.3%)	2 (5.9%)	5 (20.0%)	6 (19.4%)	10 (23.3%)	52 (17.3%)
Missing	2	0	0	2	0	0	0	2	0	1	7
Is this a children’s toothpaste? Parents, n (%)											
No	4 (13.3%)	4 (11.4%)	7 (31.8%)	6 (16.7%)	3 (12.5%)	4 (19.0%)	2 (5.9%)	6 (24.0%)	9 (29.0%)	5 (11.4%)	50 (16.6%)
Yes	25 (83.3%)	30 (85.7%)	13 (59.1%)	28 (77.8%)	17 (70.8%)	16 (76.2%)	32 (94.1%)	17 (68.0%)	20 (64.5%)	38 (86.4%)	236 (78.1%)
Don’t know	1 (3.3%)	1 (2.9%)	2 (9.1%)	2 (5.6%)	4 (16.7%)	1 (4.8%)	0 (0.0%)	2 (8.0%)	2 (6.5%)	1 (2.3%)	16 (5.3%)
Missing	2	0	0	1	0	0	0	2	0	0	5
Has the child ever been to the dentist? Parents, n (%)											
Once in a year	6 (20.0%)	14 (40.0%)	9 (40.9%)	7 (18.9%)	9 (37.5%)	2 (10.0%)	12 (35.3%)	5 (18.5%)	11 (35.5%)	14 (31.8%)	89 (29.3%)
Twice in a year	7 (23.3%)	5 (14.3%)	2 (9.1%)	5 (13.5%)	4 (16.7%)	3 (15.0%)	6 (17.6%)	4 (14.8%)	7 (22.6%)	6 (13.6%)	49 (16.1%)
More than twice in a year	14 (46.7%)	13 (37.1%)	9 (40.9%)	24 (64.9%)	10 (41.7%)	14 (70.0%)	14 (41.2%)	14 (51.9%)	9 (29.0%)	19 (43.2%)	140 (46.1%)
Never	3 (10.0%)	3 (8.6%)	2 (9.1%)	1 (2.7%)	1 (4.2%)	1 (5.0%)	2 (5.9%)	4 (14.8%)	4 (12.9%)	5 (11.4%)	26 (8.6%)
Missing	2	0	0	0	0	1	0	0	0	0	3
Reason for first dental visit (parents), n (%)											
Tooth pain	4 (14.8%)	3 (9.4%)	4 (20.0%)	3 (8.6%)	6 (26.1%)	0 (0.0%)	4 (12.5%)	3 (13.0%)	7 (25.9%)	6 (15.4%)	40 (14.3%)
Tooth trauma	1 (3.7%)	1 (3.1%)	1 (5.0%)	3 (8.6%)	0 (0.0%)	2 (9.5%)	3 (9.4%)	1 (4.3%)	1 (3.7%)	1 (2.6%)	14 (5.0%)
Tooth malposition	3 (11.1%)	5 (15.6%)	1 (5.0%)	5 (14.3%)	6 (26.1%)	3 (14.3%)	4 (12.5%)	0 (0.0%)	2 (7.4%)	5 (12.8%)	34 (12.2%)
Control	19 (70.4%)	23 (71.9%)	14 (70.0%)	24 (68.6%)	11 (47.8%)	16 (76.2%)	21 (65.6%)	19 (82.6%)	17 (63.0%)	27 (69.2%)	191 (68.5%)
Missing	5	3	2	2	1	0	2	4	4	5	28
Has the child ever had cavities? Parents, n (%)											
No	14 (46.7%)	20 (57.1%)	9 (40.9%)	26 (70.3%)	10 (41.7%)	13 (61.9%)	16 (47.1%)	17 (63.0%)	16 (51.6%)	16 (36.4%)	157 (51.5%)
Yes	16 (53.3%)	15 (42.9%)	12 (54.5%)	11 (29.7%)	14 (58.3%)	8 (38.1%)	18 (52.9%)	10 (37.0%)	15 (48.4%)	27 (61.4%)	146 (47.9%)
Don’t know	0 (0.0%)	0 (0.0%)	1 (4.5%)	0 (0.0%)	0 (0.0%)	0 (0.0%)	0 (0.0%)	0 (0.0%)	0 (0.0%)	1 (2.3%)	2 (0.7%)
Missing	2	0	0	0	0	0	0	0	0	0	2
Has the child ever lost teeth due to cavities? Parents, n (%)											
No	27 (90.0%)	31 (88.6%)	14 (63.6%)	35 (94.6%)	20 (83.3%)	20 (95.2%)	33 (100.0%)	25 (92.6%)	29 (93.5%)	38 (86.4%)	272 (89.5%)
Yes	3 (10.0%)	4 (11.4%)	8 (36.4%)	2 (5.4%)	4 (16.7%)	1 (4.8%)	0 (0.0%)	2 (7.4%)	2 (6.5%)	5 (11.4%)	31 (10.2%)
Don’t know	0 (0.0%)	0 (0.0%)	0 (0.0%)	0 (0.0%)	0 (0.0%)	0 (0.0%)	0 (0.0%)	0 (0.0%)	0 (0.0%)	1 (2.3%)	1 (0.3%)
Missing	2	0	0	0	0	0	1	0	0	0	3
Has the child lost baby teeth due to tooth decay? Parents, n (%)											
No	27 (90.0%)	31 (88.6%)	15 (68.2%)	35 (94.6%)	20 (83.3%)	20 (95.2%)	33 (100.0%)	25 (92.6%)	29 (93.5%)	39 (88.6%)	274 (90.1%)
Yes	3 (10.0%)	4 (11.4%)	7 (31.8%)	2 (5.4%)	4 (16.7%)	1 (4.8%)	0 (0.0%)	2 (7.4%)	2 (6.5%)	5 (11.4%)	30 (9.9%)
Missing	2	0	0	0	0	0	1	0	0	0	3
Has your child lost permanent teeth through decay? Parents, n (%)											
No	30 (100.0%)	35 (100.0%)	21 (95.5%)	37 (100.0%)	22 (91.7%)	21 (100.0%)	33 (100.0%)	27 (100.0%)	31 (100.0%)	44 (100.0%)	301 (99.0%)
Yes	0 (0.0%)	0 (0.0%)	1 (4.5%)	0 (0.0%)	2 (8.3%)	0 (0.0%)	0 (0.0%)	0 (0.0%)	0 (0.0%)	0 (0.0%)	3 (1.0%)
Missing	2	0	0	0	0	0	1	0	0	0	3
Has the child lost both baby and permanent teeth through decay? Parents, n (%)											
No	30 (100.0%)	35 (100.0%)	22 (100.0%)	37 (100.0%)	22 (91.7%)	21 (100.0%)	33 (100.0%)	27 (100.0%)	31 (100.0%)	44 (100.0%)	302 (99.3%)
Yes	0 (0.0%)	0 (0.0%)	0 (0.0%)	0 (0.0%)	2 (8.3%)	0 (0.0%)	0 (0.0%)	0 (0.0%)	0 (0.0%)	0 (0.0%)	2 (0.7%)
Missing	2	0	0	0	0	0	1	0	0	0	3

**Table 9:** DMFT/dmft and IP measures.

	School										
	Monza Buonarroti (n = 32)	Monza Manzoni (n = 35)	Monza Citterio (n = 22)	Desio Collegio Di Rosa (n = 37)	Madone (n = 24)	Merate Sartirana (n = 21)	Villa D’Adda (n = 34)	Mapello (n = 27)	Bonate Sotto (n = 31)	Brembate Sopra (n = 44)	Total (n = 307)
Have you ever been to the dentist? n (%)											
No	2 (6.3%)	2 (5.7%)	2 (9.1%)	4 (10.8%)	2 (8.3%)	1 (4.8%)	1 (2.9%)	4 (14.8%)	4 (12.9%)	6 (13.6%)	28 (9.1%)
Yes	30 (93.8%)	33 (94.3%)	20 (90.9%)	33 (89.2%)	22 (91.7%)	20 (95.2%)	33 (97.1%)	23 (85.2%)	27 (87.1%)	38 (86.4%)	279 (90.9%)
rate_dmft_all ^a^											
N (missing)	32 (0)	35 (0)	22 (0)	37 (0)	24 (0)	21 (0)	34 (0)	27 (0)	31 (0)	44 (0)	307 (0)
Mean (SD)	0.0 (0.04)	0.0 (0.06)	0.0 (0.07)	0.0 (0.03)	0.0 (0.08)	0.0 (0.04)	0.1 (0.07)	0.0 (0.03)	0.0 (0.09)	0.0 (0.06)	0.0 (0.06)
rate_dmft_per ^b^											
N (missing)	32 (0)	35 (0)	22 (0)	37 (0)	24 (0)	21 (0)	34 (0)	27 (0)	31 (0)	44 (0)	307 (0)
Mean (SD)	0.0 (0.07)	0.1 (0.09)	0.1 (0.13)	0.0 (0.06)	0.1 (0.16)	0.0 (0.07)	0.1 (0.11)	0.0 (0.06)	0.1 (0.17)	0.1 (0.10)	0.1 (0.11)
DMFT/dmft											
N (missing)	32 (0)	35 (0)	22 (0)	37 (0)	24 (0)	21 (0)	34 (0)	27 (0)	31 (0)	44 (0)	307 (0)
Mean (SD)	0.4 (0.91)	0.7 (1.24)	0.8 (1.53)	0.2 (0.84)	1.2 (1.90)	0.4 (0.92)	1.1 (1.31)	0.4 (0.74)	0.8 (1.47)	0.9 (1.21)	0.7 (1.26)
DMFT_dico ^c^											
N (missing)	32 (0)	35 (0)	22 (0)	37 (0)	24 (0)	21 (0)	34 (0)	27 (0)	31 (0)	44 (0)	307 (0)
Mean (SD)	0.2 (0.40)	0.4 (0.49)	0.3 (0.46)	0.1 (0.28)	0.4 (0.50)	0.2 (0.40)	0.6 (0.50)	0.3 (0.45)	0.3 (0.46)	0.5 (0.50)	0.3 (0.47)
Teeth											
N (missing)	32 (0)	35 (0)	22 (0)	37 (0)	24 (0)	21 (0)	34 (0)	27 (0)	31 (0)	44 (0)	307 (0)
Mean (SD)	22.8 (2.24)	22.3 (2.42)	22.7 (1.78)	22.3 (3.53)	23.3 (2.09)	23.3 (1.20)	21.7 (4.61)	23.1 (2.71)	23.0 (2.58)	22.5 (4.30)	22.6 (3.11)
Deciduous teeth											
N (missing)	32 (0)	35 (0)	22 (0)	37 (0)	24 (0)	21 (0)	34 (0)	27 (0)	31 (0)	44 (0)	307 (0)
Mean (SD)	9.4 (2.42)	8.3 (3.11)	9.4 (3.37)	7.8 (3.64)	8.7 (4.73)	9.7 (2.87)	7.2 (4.38)	10.6 (2.15)	9.9 (3.31)	9.6 (3.69)	9.0 (3.56)
Permanent teeth											
N (missing)	32 (0)	35 (0)	22 (0)	37 (0)	24 (0)	21 (0)	34 (0)	27 (0)	31 (0)	44 (0)	307 (0)
Mean (SD)	13.4 (2.12)	13.9 (3.24)	13.4 (4.50)	14.5 (4.80)	14.6 (4.57)	13.6 (2.46)	14.5 (4.08)	12.5 (1.72)	13.1 (3.35)	12.9 (3.69)	13.7 (3.64)
Plaque index (%)											
N (missing)	32 (0)	35 (0)	22 (0)	37 (0)	24 (0)	21 (0)	34 (0)	27 (0)	31 (0)	44 (0)	307 (0)
Mean (SD)	0.8 (0.28)	0.9 (0.24)	0.7 (0.29)	0.8 (0.26)	0.7 (0.31)	0.6 (0.28)	0.8 (0.23)	0.9 (0.22)	0.8 (0.27)	0.8 (0.23)	0.8 (0.26)

^a^ DMFT/number of teeth; ^b^ DMFT/permanent teeth; ^c^ 1 = more than one decayed tooth, 0 = if not.

**Table 10:** Fixed-effect estimates from the mixed-effects logistic regression model for DMFT/dmft ≥ 1 (random intercept for schools) with selected variables. Coefficients are reported on the logit scale with standard errors. Var(u0j) denotes the estimated variance of the random intercept.

Effect	Estimate	Standard Error
Intercept	−1.89	0.57
Plaque index (%)	2.71	0.65
Snack 3	−1.89	0.66
K1	−0.56	0.35
K12	1.25	0.54
IG12	−2.20	0.32
Var ($u_{0j}$)	0.16	0.17

## Data Availability

The datasets used and/or analyzed during the current study are available from the corresponding author on reasonable request due to privacy.

## Appendix A

Box A1 Measures of model performance [44]. Discrimination The c-index provides an indication of how well the model can discriminate between patients who experience the event and patients who do not: Perfect discrimination, c = 1. Poor or no discrimination, c = 0.5. The c-index is the probability of concordance between outcomes and predictions. For binary outcomes this is the probability that a randomly chosen individual with the event has a higher predicted probability than a randomly chosen individual without the event. It is identical to the area under the receiver operating characteristic (ROC) curve, i.e., AUC. Calibration Calibration assesses whether the average prediction effects are correct by evaluating the intercept and slope of the calibration line (obtained by regressing the outcome on the logit of the predicted probabilities). Calibration intercept: measures systematic over- or underestimation (ideal value = 0). Calibration slope: measures overfitting or underfitting (ideal value = 1; <1 suggests overfitting, >1 suggests underfitting).

**Table A1:** Demographic characteristics of excluded participants.

	Total (n = 51)
Child Age	
n	47
Mean (SD)	9.3 (0.47)
Median	9.0
Range	9.0, 10.0
Gender, n (%)	
Male	23 (46.9%)
Female	26 (53.1%)
Not answered	2
Child nationality, n (%)	
Foreign	3 (13.0%)
Italian	20 (87.0%)
Not answered	28
Parents’ nationality, n (%)	
Both foreign	10 (38.5%)
Both Italian	16 (61.5%)
Not answered	25
Parents’ education, n (%)	
Middle school or less	4 (16.0%)
High school	15 (60.0%)
Degree	6 (24.0%)
Not answered	26
Classification of weight status according to Cole class, n (%)	
Underweight	2 (8.0%)
Normal weight	17 (68.0%)
Overweight	4 (16.0%)
Obese	2 (8.0%)
Not answered	26

**Table A2:** Number of times a variable has been selected in 500 bootstrap replicates. IP = plaque index; LIV_ISTR = parent education; dent_new = have you ever been to the dentist?; zBMI = standardized BMI; zCOLE = standardized Cole.

Variable	Count
IG12	500
IP	500
SNACK 3	500
K12	359
K1	282
SNACK 14	260
SNACK 6	228
SNACK 7	187
zBMI	177
LIV_ISTR	173
dent_new	158
zCOLE	122
IG7	121

**Table A3:** Number of times a model has been selected in 500 bootstrap replicates. IP = plaque index; LIV_ISTR = parent education; dent_new = have you ever been to the dentist?; zBMI = standardized BMI; zCOLE = standardized Cole.

Selected Mode	Count
zBMI, zCOLE, IP_new, LIV_ISTR, SNACK_3, SNACK_6, SNACK_7, SNACK_14, K1, K12, IG7, IG12, dent_new	28
IP_new, SNACK_3, SNACK_14, K12, IG12	15
IP_new, SNACK_3, SNACK_6, SNACK_14, K12, IG12	11
IP_new, SNACK_3, SNACK_6, K1, K12, IG12	10
IP_new, SNACK_3, K1, K12, IG12	9
zBMI, IP_new, SNACK_3, K1, K12, IG12	9
IP_new, SNACK_3, SNACK_14, IG12	8
IP_new, SNACK_3, SNACK_7, SNACK_14, K1, K12, IG12	7
IP_new, SNACK_3, K12, IG12	6
IP_new, SNACK_3, K1, K12, IG12, dent_new	6
IP_new, SNACK_3, SNACK_14, K12, IG12, dent_new	6
IP_new, SNACK_3, SNACK_6, SNACK_14, IG12	6
IP_new, LIV_ISTR, SNACK_3, IG12, dent_new	5
IP_new, SNACK_3, SNACK_6, SNACK_14, K1, K12, IG12	5
IP_new, SNACK_3, SNACK_6, SNACK_7, K1, K12, IG12	5
IP_new, SNACK_3, SNACK_6, SNACK_7, SNACK_14, K1, K12, IG12	5
zBMI, IP_new, SNACK_3, SNACK_6, SNACK_14, K1, K12, IG12	5
zBMI, zCOLE, IP_new, SNACK_3, K1, K12, IG12	5
IP_new, LIV_ISTR, SNACK_3, SNACK_14, K12, IG12	4
IP_new, SNACK_3, IG12	4
